# Is it necessary to split nitrogen fertilization for winter wheat? On-farm research on Luvisols in South-West Germany

**DOI:** 10.1017/S0021859614000288

**Published:** 2014-05-01

**Authors:** R. SCHULZ, T. MAKARY, S. HUBERT, K. HARTUNG, S. GRUBER, S. DONATH, J. DÖHLER, K. WEIß, E. EHRHART, W. CLAUPEIN, H.-P. PIEPHO, C. PEKRUN, T. MÜLLER

**Affiliations:** 1 Institute of Crop Science: Fertilization and Soil Matter Dynamics (340i), University of Hohenheim, D-70593 Stuttgart, Germany; 2 Nuertingen-Geislingen University, Agronomy, D-72622 Nürtingen, Germany; 3 Institute of Crop Science: Bioinformatics (340c), University of Hohenheim, D-70593 Stuttgart, Germany; 4 Institute of Crop Science: Agronomy (340a), University of Hohenheim, D-70593 Stuttgart, Germany; 5 Landratsamt Tübingen, 72072 Tübingen, Germany; 6 Regierungspräsidium Tübingen, 72072 Tübingen, Germany

## Abstract

Mineral nitrogen (N) fertilization in cereals is commonly split into three or four applications. In order to simplify N fertilization, a single N application either broadcast or placed on the soil surface was compared to conventionally split fertilization for winter wheat (*Triticum aestivum* L.). The 4-year experiment (2007–2010) was performed using a participatory approach on farmers’ fields on deep loamy soils (Luvisols) in South-West Germany.

Grain yield and crude protein contents differed only slightly or not at all between treatments including different N fertilizer types (calcium ammonium nitrate, urea ammonium nitrate solution, urea) and application techniques (broadcast, placed). Furthermore, no differences were found for the yield components ears/m^2^ and thousand grain weight. Inorganic N in the soil profile after harvest was generally below 40 kg N/ha and did not differ between treatments. In the area where N was placed, mineral N was depleted during the vegetation period.

At the experimental sites a single N application in the period between tillering and stem elongation was sufficient to achieve high yield and quality of winter wheat without increased risk of nitrate leaching. This finding was independent of the method of application or the type of fertilizer.

## INTRODUCTION

Increasing farm sizes with low manpower, increasing energy costs and ecological problems due to nitrogen (N) losses call for improved and simplified N fertilization. At present, management guidelines for cereals in Europe and the USA include recommendations for split application of mineral N. Splitting the fertilization into three or four doses is supposed to support specific yield components depending on the time of application and the corresponding stages of plant development (Hamid 1972; Alcoz *et al.* 1993). Furthermore, a split application is thought to avoid lodging and N losses by leaching (Gerwing *et al.* 1979; Kanwar *et al.* 1988; Varshney *et al.* 1993) because N application and N uptake are balanced both in time and amount.

However, other research indicates that weather conditions and total N supply are more important for yield and crude protein contents of cereals than splitting and the timing of applications (Fischbeck *et al.* 1990; Alcoz *et al.* 1993; Maidl *et al.* 1996). The effect of a split-N application on yield and quality of wheat is low if the total amount of N is sufficient (Müller *et al.* 1991) and the last N application might not be taken up effectively by the plants, particularly under dry weather conditions in May and June, at the heading stage (Hartman & Nyborg 1989).

According to Fox *et al.* (1986) the timing of N application is an important factor for increasing N use efficiency (NUE). Nitrogen use efficiency is increased if N fertilization is restrained until tillering, but ample in the growth stages (GS) 30–39 (Zadoks *et al.* 1974; Maidl *et al.* 1998; Sticksel *et al.* 2000). It is thought that a limited N application before tillering avoids disproportionate side shoot production and excessive water consumption. Further, the risk of lodging as a result of rapid stem elongation is lower. However, the yield potential of modern winter wheat genotypes is mainly attributed to a higher partitioning of biomass to the grains, resulting in short stems (Austin *et al.* 1980) and thus reducing the risk of lodging compared to old genotypes.

Decades ago, many cultivars could be characterized as ‘density types’ or ‘ear types’ producing high yields either by a high density of ear-bearing stems (density types) or by a high number of spikelets/ear and a high thousand grain weight (ear type). Consequently, split-N fertilization was aimed at the promotion of single yield components depending on the variety. Modern cultivars, however, can be characterized as ‘compensation types’, where later-developing yield components (e.g. spikelets/ear) can compensate for limitations during the growth of yield components developed earlier (e.g. ear bearing stems/m^2^), as it was decades ago. This is also visible in the German Descriptive Variety Lists, where the expression of yield components is characterized on a scale between 1 and 9 for each yield component. Extremes are very rare in the current list and all wheat varieties are characterized by medium values between 4 and 6 for at least two of the three yield components (Bundessortenamt 2012).

To simplify N fertilization, a reduction to one or two broadcast N applications may be an alternative to a traditional N application split into three or four doses. A single broadcast application would offer the advantage that only one pass over the field is needed and that no special application techniques are required, e.g. fertilizer placement.

An alternative to the split-N application is the placed ammonium (NH_4_^+^) application, as a modification of the controlled uptake long-term ammonium nutrition (CULTAN)-fertilization described by Sommer (2000, 2005). The whole N amount is applied in one single dose in spring, either directly injected into the soil or placed on the soil surface as depots or depot bands with high NH_4_^+^ concentration. Typically, this application is done much later than the traditional first application. This forces the plant to invest in root growth, facilitating nutrient and water uptake at a later stage. As an alternative, a part of the NH_4_^+^ may be replaced by urea, which is assumed to mineralize quickly to NH_4_^+^. A diffusion zone develops concentrically around the depot, from which NH_4_^+^ and – after nitrification (and following diffusion and mass flow) – nitrate (NO_3_^−^) can be taken up by plant roots (Schittenhelm & Menge-Hartmann 2006; Menge-Hartmann & Schittenhelm 2008). A high share of NH_4_^+^ uptake in contrast to the usually dominating NO_3_^−^ uptake may affect the distribution of assimilates within the plant, the phytohormone production and the intensity of growth of different organs (Sommer & Scherer 2009). An injection of N fertilizer results in lower gaseous N losses and provides an appropriate amount of N under dry conditions, particularly under a soil surface mulch layer in conservation tillage or no-till systems (Liu *et al.* 2006).

To obtain direct and bilateral transfer of knowledge and methodologies, the current investigation was conducted as a joint approach between farmers, local extension service and the university. To address farmers’ needs, the main object was to investigate N fertilizing strategies in winter wheat under normal farming practice on farmers’ fields. This is in contrast to most of the studies mentioned above, which were based on on-station experiments. Studies on farmers’ fields are termed ‘on-farm experiments’ and aim to obtain practice-orientated results.

Soil nutrients, plot size, equipment, etc. are the main differences between fields within farms and between farms, causing great variations. Hence, for statistical analysis of on-farm experiments, the same requirements as for on-station experiments need to be fulfilled: randomization, replication, blocking (Piepho *et al.* 2011).

Thus, the aim of the present study was to investigate strategies for a simplified N fertilization in winter wheat under practice-related conditions on farmers’ fields. Besides the traditional three-split applications the alternative strategies tested were one or two applications of broadcast N and a single placed NH_4_^+^ fertilization. Following the idea of Sommer (2000, 2005), the single application and the first application of the two times split application should be late, at the end of tillering/beginning of shooting (GS 27–32), when the second application was done for the three times split application.

The following hypotheses, related to the site conditions of the current investigation, were tested: (1) a reduction in the number of N broadcast applications to winter wheat with a late (first) N application does not affect yield and quality; (2) a reduction of the number of N broadcast applications to winter wheat with a late (first) N application does not increase the risk for NO_3_^−^ leaching losses; and (3) compared to a single broadcast application of N, a single placed depot application of NH_4_^+^ – and urea-dominated fertilizers leads to higher yields and to a reduced risk for NO_3_^−^ leaching.

## MATERIALS AND METHODS

### Study sites

The investigation was performed over 4 years in two rural districts, Tübingen (48°24′–48°28′N, 8°50′–9°05′E) and Biberach (48°01′–48°08′N, 9°26′–9°46′E), Baden-Württemberg, South-West Germany. Four agricultural farms in 2007, six in 2008, 11 in 2009 and 11 in 2010 took part with one or more fields in the intended fertilization experiments. Each field included three to five plots, and, thus, the same number of fertilization treatments. The size of one plot was *c*. 100 m long×*c*. 12–16 m wide, depending on the working width of the farm machinery used and the individual field conditions.

The study sites varied slightly in terms of climatic conditions and altitude above sea level. The experimental fields are characterized as follows: (1) Tübingen: 450 m a.s.l.; average annual temperature: 9–10 °C; soil textures: silty loam and clayey loam; (2) Biberach: 550 m a.s.l.; average annual temperature: 8–9 °C; soil textures: silty loam and sandy loam. Soil types on 12 farms in both areas were Haplic Luvisols, on three farms Gleyic Luvisols and on two farms Calcic Cambisols. The average annual precipitation (1960–1990) was similar in both rural districts and accounted for 700 (Tübingen) – 800 mm (Biberach). The long-term average climatic water balance (1960–1990) for the hydrological summer half-year was 0–100 mm in Tübingen and 100–200 mm in Biberach (Wasser- und Bodenatlas Baden-Württemberg 2012). Since average temperatures and thus evaporation increased in the last two decades and dry springs appeared more often (see the later section on weather conditions), so the risk of nitrate leaching during vegetation period from loamy soils was assumed to be low.

### Farmer participatory approach

The experimental setup and fertilization treatments were developed jointly with participating farmers and local extension services, during workshops prior to the start of the experiments. Data from the first experimental year were presented to the farmers as a pre-requisite for modifications in the experimental design of the following years. The final results were presented to the farmers and discussed together after each year.

More than half of the participating farms had livestock. All farms practised conservation soil tillage (no soil inversion by mouldboard ploughing) and none of them used plant growth regulators to avoid lodging. According to the recommendations of the official extension services, available plant nutrients [phosphorus (P), potassium (K), magnesium (Mg) and sulphur (S)] in the soil were at a medium level and thus not expected to limit plant production.

Winter high-protein wheat cultivars chosen by farmers in the 4 years are characterized mainly as compensation types, some with slight tendencies towards plant density or ear types. The preceding crops were oilseed rape, sugar beet, potatoes, pea or winter wheat and, on one field only, strawberries.

### Fertilizer treatments

Fertilizer types, application techniques, number of applications, application times and number of replications used in the field experiments are shown in Table 1. At each field, selected treatments were arranged, representing one (incomplete) block (see statistics below). Control plots (small zero fertilization windows) without N fertilization were partially installed, but are not included in the statistical analysis.

**Table 1.** Fertilizer types, application technique, number of applications, application times and number of fields (replicates)

Treatment No.	Fertilizer types/application technique	No. of applications	Application time (GS)*	Number of fields (replicates)			
				2007	2008	2009	2010
1	Calcium ammonium nitrate (CAN)/broadcast	3	GS 21–25, 27–32, 47–51	4	3	9	11
2	CAN/broadcast	2	GS 27–32, 47–51			4	8
3	CAN/broadcast	1	GS 27–32	6	4	9	11
4	Urea/broadcast	1	GS 27–32	5	4	4	1
5	Urea ammonium nitrate solution (UAN)/placed (banded)	1	GS 27–32	7	6	5	6

* Growth stage according to the Zadoks scale (Zadoks *et al*. 1974).

The amount of fertilizer N applied on each field was calculated in line with recommendations of the official extension service. This takes into account the expected N demand considering the expected grain yield and crude protein content. The inorganic N already available in soil, sampled in early spring in the 0–90 cm soil layer, was subtracted from the expected N demand, as well as the assumed N delivery from soil during the cultivation period, long-term manure application and plant residues of pre-crops and intermediate crops (Düngeverordnung 2009). The fertilizer N amount for an individual experimental field was always the same for all treatments. The fertilizer N amount was usually in the range 200±20 kg N/ha and only exceptionally lower or higher. As a result, the total N-supply was in the range of 180–260 kg N/ha.

Broadcast fertilizer application was achieved using farmers’ broadcast or pneumatic spreaders. Broadcast spreaders were used half-sided so that a single plot was fertilized by an exact overlap of two crossings. Placed surface application (band application) of urea ammonium nitrate solutions was carried out using pesticide sprayers in combination with tracking hoses.

### Weather conditions

Climatic conditions in South-West Germany have changed in recent decades, moving towards higher average annual temperatures and more extreme weather situations including dry conditions in spring and more thunderstorms with heavy rainfall in summer. The average annual temperature in the area of the field experiments at Tübingen increased from 8·8 °C (1961–1990) to 9·9 °C (1991–2010) (Institute of Physics and Meteorology, University of Hohenheim). Therefore, it is worth comparing the weather conditions during the growing season of winter wheat with the mean weather data of the last two decades (1991–2010) (Table 2).

**Table 2.** Weather conditions 2007–2010 during the main growing season and deviation from mean values of the last two decades 1991–2010. (Institute of Physics and Meteorology, University of Hohenheim)

Year	Month	Temperature (°C)	Deviation from long-term average (°C)	Precipitation (mm)	Deviation from long-term average (mm)
2007	March	6·3	+0·6	57·2	+2·9
	April	13·9	+4·2	0·5	−40·8
	May	15·5	+1·4	116·8	+33·5
	June	18·0	+0·8	131·3	+46·6
	July	18·0	−1·0	85·4	−5·0
2008	March	5·1	−0·6	53·2	−1·1
	April	8·4	−1·3	68·0	+26·7
	May	16·0	+1·9	96·4	+13·1
	June	18·0	+0·8	93·9	+9·2
	July	18·8	−0·3	64·5	−25·9
2009	March	4·7	−1·0	82·6	+28·3
	April	12·6	+2·9	17·7	−23·6
	May	15·3	+1·2	129·1	+45·8
	June	16·5	−0·7	76·5	−8·2
	July	18·8	−0·3	140·3	+49·9
2010	March	4·9	−0·8	28·0	−26·3
	April	10·1	+0·4	7·4	−33·9
	May	11·4	−2·7	83·8	+0·5
	June	17·5	+0·3	70·4	−14·3
	July	20·8	+1·7	99·0	+8·6

Weather conditions during the cultivation of winter wheat in the 4 years of field experiments were characterized by extremes. In 3 out of the 4 years (2007, 2009, 2010), April was dry and relatively warm, followed by high precipitation in May in 2007 and 2009. There is a trend towards a dry spring in South-West Germany, whereas precipitation as well as temperature in the remaining months was highly variable compared with the average of 1991–2010.

### Data collection

#### Plant analyses

Harvesting of the individual treatments was performed by combine harvesters with integrated grain yield recording. In addition, in 2007 and 2008 yield components (spikes/m^2^, grains/ear, thousand grain weight) were determined by sampling plants in five (2007) or three sampling points (2008) per treatment in each field, each of which had an area of 0·28 m^2^.

Plant samples were oven dried (60 °C) to constant weight and nitrogen concentrations were analysed with a *C*/*N* auto-analyser (‘Elementar’, model ‘vario Max CN’, Hanau, Germany). Crude protein content was determined by multiplying N concentration by a factor of 5·7 (Teller 1932).

#### Soil analyses

Soil samples for determination of inorganic N (NH_4_^+^ and NO_3_^−^) were taken from each field in spring before N fertilization (0–90 cm) and from each plot after harvest (0–90 cm) (VDLUFA 2007). Furthermore, soil samples (0–30 cm) were taken from the centre of an application band of urea ammonium nitrate solution during the vegetation period to determine the concentration of NO_3_^−^ and NH_4_^+^ in the fertilizer band. Placement bands were labelled in the field immediately after application using plastic sticks to ensure correct soil sampling. Soil samples of each plot and at each time were taken in triplicate. Samples were analysed for inorganic N with a continuous flow analyser (AutoAnalyzer 3, Bran + Luebbe/SEAL Analytical Norderstedt, Germany).

### Statistics

The current investigation of N fertilizing strategies in winter wheat was conducted under normal farming practices. A fertilizing strategy (treatment) was defined as a combination of a fertilizer treatment (*F*) and an application frequency (*A*). Farmers were allowed to test additional individual fertilizing strategies as long as they also tested some of the fertilizing strategies of interest to the scientific partners (co-treatments). On every field, thus, core treatments appeared together with individual treatments tested on some fields only. The most obvious design for this purpose is the incomplete block design, defining each field as an incomplete block. The different treatments tested on that field are randomized (Piepho *et al.* 2011). A pre-requisite for this design is that each block contains at least one treatment which occurs in at least one other block, so that all blocks are connected. The values of yield and crude protein contents were collected in years 2007–2010 on all fields and an ANOVA could be conducted. Soil mineral N and yield components, such as ears/m^2^, grains/ear and thousand grain weight, were collected in the first 2 years but on some fields only, resulting in a poor database. Therefore, only descriptive statistics were used.

On-farm field trial data from 4 years were analysed separately for each year and jointly for all years. The yield data were collected from 33 fields at 15 farms and the crude protein data from 34 fields at 16 farms. Each field on the farms was used only once over the 4-year period. The trials comprised nine fertilizer treatments (*F*) and three application frequencies (*A*). Among the possible treatments formed from factors *F* and *A*, up to 14 were tested. Among these 14 combinations, the main focus were five combinations (Table 1), i.e. on calcium ammonium nitrate (CAN) applied once, twice or three times as well as on urea and urea ammonium nitrate solution (UAN) applied once.

For yield and crude protein, all available combinations were used for statistical analyses. The number of treatments tested per field varied between two and six. Due to the design of the on-farm trials, the data were highly unbalanced (Table 3).

**Table 3.** Overview of year×farm×field×treatment combinations available in the dataset. Crosses identify the available combinations

Farm	Field	Year	CAN			Urea	UAN
			1 Appl.*	2 Appl.	3 Appl.	1 Appl.	1 Appl.
1	1	2010	x	x	x		x
2	2	2010	x		x		x
3	3	2007			x	x	x
3	4	2008				x	x
3	5	2010	x	x	x		x
4	6	2010	x	x	x		
5	7	2010	x	x	x		
6	8	2008	x		x	x	
6	9	2009	x		x	x	x
6	10	2010	x		x	x	x
7	11	2009	x	x	x		
7	12	2009	x	x	x		
7	13	2010	x	x	x		
8	14	2009	x		x		x
8	15	2010	x		x		
9	16	2007			x	x	x
9	17	2007				x	x
9	18	2010	x	x	x		x
10	19	2008				x	x
10	20	2009	x		x	x	x
11	21	2009	x	x	x		
12	22	2007	x		x	x	x
12	23	2007	x		x	x	x
12	24	2009	x		x	x	
13	25	2007	x		x	x	x
13	26	2007	x		x	x	x
13	27	2008	x		x		x
13	28	2009	x		x		
14	29	2009	x		x	x	
14	30	2010	x	x	x		
15	31	2009					x
16	32	2009					x
17	33	2008			x†		x†
18	34	2008			x		x
18	35	2010	x	x	x		x
19	36	2008				x	x

* Application.

† Only data for crude protein were available for farm 17, field 11.

The mixed model for a single year was:(1)
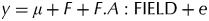
where *y* is the dependent variable yield or crude protein; *μ* the overall mean; *F* is the main effect of fertilizer; *F.A* is the nested effect of application frequency (*A*) within fertilizer; FIELD is the main effect for field and *e* is a residual error. The colon separates fixed effects, listed first, from random effects. The mixed model across years was:(2)
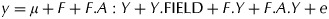
where fixed effects are as defined for Eqn (1) and random effects now include random interaction effects with year (*Y*) as well as a field effect nested within years (*Y*.FIELD). Wald-type *F*-tests of fixed effects were performed, adjusting the denominator d.f. using the Kenward–Roger method. The Tukey test, which controls the family-wise type I error rate, was used for mean comparisons among the five treatments. The adjusted means shown in figures are those from the model across years containing all combinations of fertilizer and application frequency. Due to the unbalanced nature of the data, letter displays were generated using the method of Piepho (2012).

## RESULTS

Wald-type *F*-tests for fixed effects as well as variance component estimates for random effects are reported in Table 4. The adjusted means are shown in Table 5.

**Table 4.** *F*-tests (fixed effects) and variance component estimates (random effects) for yield and crude protein for analyses across years and per year. For a single year there is no *Y*×Field effect but only a FIELD effect, but these effects are reported in a single column

	Fixed effects (*F*-tests)				Random effects (variance components)				
	Fertilizer		Fertilizer application frequency		Year	*Y*.FIELD/FIELD	*F.Y*	*F.A.Y*	Error term*
	*F* value	*P* value	*F* value	*P* value					
Yield 2007–2010	0·89	NS	0·78	NS	100·76	132·45	0	0	9·26
Yield 2007	0·03	NS	0·37	NS	–	78·86	–	–	13·44
Yield 2008	1·08	<0·05	0·62	NS	–	54·38	–	–	13·54
Yield 2009	0·10	NS	0·80	NS	–	225·60	–	–	8·91
Yield 2010	0·87	NS	0·63	NS	–	86·03	–	–	12·73
Crude protein 2007–2010	1·58	NS	2·58	NS	1·25	0·99	0·009	0·06	0·29
Crude protein 2007	8·72	<0·01	2·22	NS	–	0·71	–	–	0·17
Crude protein 2008	0·39	NS	6·14	<0·05	–	1·06	–	–	0·33
Crude protein 2009	3·57	NS	3·21	NS	–	0·94	–	–	0·29
Crude protein 2010	2·22	NS	5·19	<0·05	–	1·09	–	–	0·32

* Residual variance of the model.

**Table 5.** Adjusted means, mean grouping by letters (±s.e.m.) for the five fertilizer treatments for yield and crude protein for analyses across years and per year

	Fertilizer	No. of Appl.	Mean				
			2007–2010	2007	2008	2009	2010
Yield	CAN	1	79·2±5·47	85·2±3·89	92·8±4·35	68·6±4·67	71·6±3·00
	CAN	2	79·5±5·5	–	–	67·7±4·99	72·7±3·10
	CAN	3	80·3±5·4	83·7±3·70	95·6±3·93a	70·0±4·67	73·3±3·00
	Urea	1	79·6±5·5	84·9±3·63	91·6±3·64a	69·6±4·84	68·3±4·95
	UAN	1	80·8±5·48	84·9±3·63	95·5±3·46	69·3±4·81	73·7±3·21
Crude Protein	CAN	1	13·0±0·62a	14·2±0·39a	11·1±0·63a	12·5±0·35ab	14·1±0·36a
	CAN	2	13·3±0·66a	–	–	12·9±0·47ab	14·5±0·38ab
	CAN	3	13·6±0·61a	13·8±0·36ab	12·5±0·51a	13·2±0·35a	14·9±0·36b
	Urea	1	12·9±0·62a	13·1±0·35b	11·8±0·51a	11·9±0·42	15·7±0·72b
	UAN	1	13·3±0·61a	13·8±0·35a	12·1±0·46a	12·6±0·41b	14·9±0·40b

* Means within a column and trait showing a common letter are not significantly different according to a Tukey test. No significant differences were found for yield.

### Grain yield

**Fig. 1.**
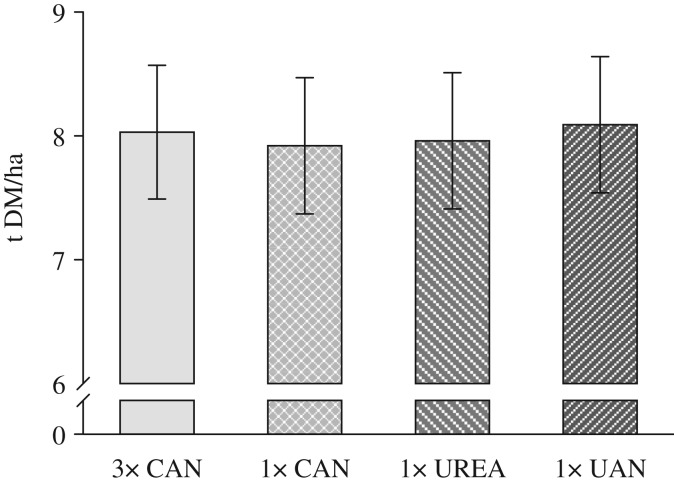
Mean grain yield of the fertilization treatments calcium ammonium nitrate (CAN), urea and urea ammonium nitrate solution (UAN) 2007–2010. Bars show s.e. of the means.

The grain yields of the different fertilization treatments including the N fertilizer types CAN, urea and UAN solution, the number of applications (3×CAN, 1×CAN, 1×urea, 1×UAN) and the different application techniques (CAN and urea broadcast; UAN placed) were not significantly different, either over the period of 4 years (Fig. 1) or in any of the 4 years (data not shown). Yields of the control plots (if existing) were typically between 0·60 and 0·65 of the other treatments. However, due to different weather conditions, the mean yield varied highly between the four years. In 2007, 2008, 2009 and 2010 the average grain yield levels of the core treatments were 8·5, 9·4, 7·0 and 7·3 t DM/ha, respectively.

Although no growth regulators were applied by farmers, no lodging occurred in any of the 4 years, not even after a single application. In some fields, winter wheat plants in the treatments with a reduced number of applications showed clear N deficiency symptoms at the end of tillering. However, *c*. 3 weeks after the late (first) application, deficiency symptoms disappeared and visual differences between the fertilization treatments could no longer be observed.

**Fig. 2.**
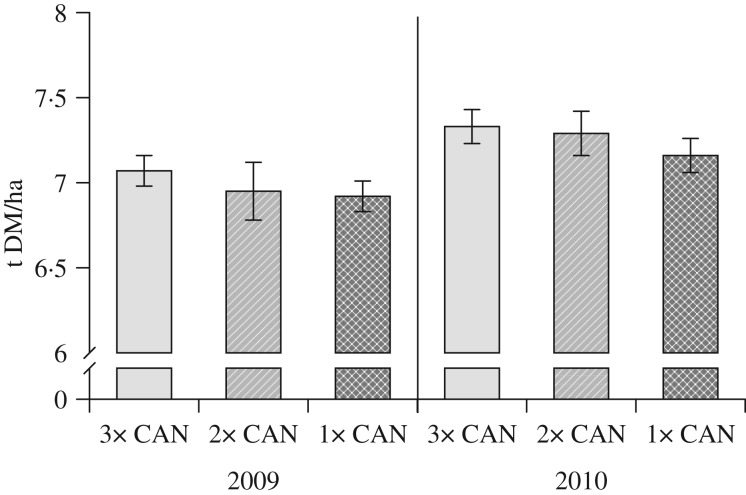
Grain yield of the split (3×, 2×) and single (1×) applications of calcium ammonium nitrate (CAN) in 2009 and 2010. Bars show s.e. of the means.

The two-time split application of CAN was included in 2009 and 2010 only. In both years, there was no difference in grain yield between this treatment and conventional N fertilization applied three times (Fig. 2).

### Crude protein

**Fig. 3.**
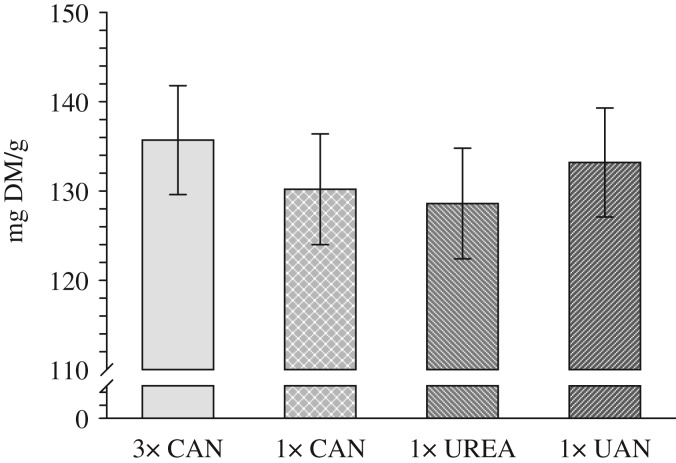
Mean crude protein content of the fertilization treatments three times (3×) and single application (1×) of calcium ammonium nitrate (CAN), and single application of urea and urea ammonium nitrate solution (UAN) 2007–2010. Bars show s.e. of the means.

**Fig. 4.**
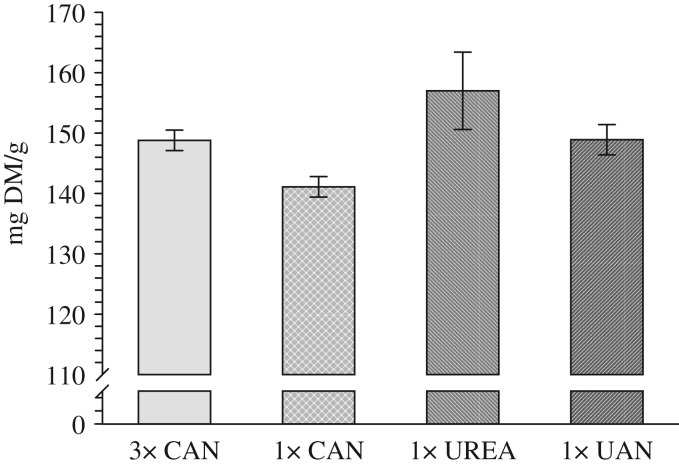
Crude protein content of the fertilization treatments three times (3×) and single application (1×) of calcium ammonium nitrate (CAN), and single application of urea and urea ammonium nitrate solution (UAN) in 2010. Bars show s.e. of the means.

There were no significant differences in crude protein contents between the treatments (conventional split application of CAN, single broadcast application of CAN or urea and single placed application of UAN solution) over a period of 4 years (Fig. 3). No significant differences were observed in the individual years either, except in 2010 which had relatively high crude protein contents in all treatments. Here, the crude protein of the single application of CAN was significantly lower (*P*<0·05) than that of the conventional split application (Fig. 4). The mean crude protein levels in 2007, 2008, 2009 and 2010 were 13·7, 11·9, 12·4 and 14·9%, respectively. In the year with highest grain yield (2008 : 9·4 t DM/ha), crude protein was lowest.

Yield components were measured in the first 2 years of the field experiments. In 2008, spikes averaged 587 ears/m^2^ with no difference between treatments. In 2007 a mean value of 39·7 grains/ear was measured, showing higher numbers for the single broadcast application of urea and the single placed application of UAN solution as compared to the conventional split application (data not shown). In contrast, in 2008 no differences appeared between these treatments. The thousand grain weight did not differ between any of the treatments in 2008. An average of 46 g dry weight per thousand grains was measured.

### Soil mineral N

**Fig. 5.**
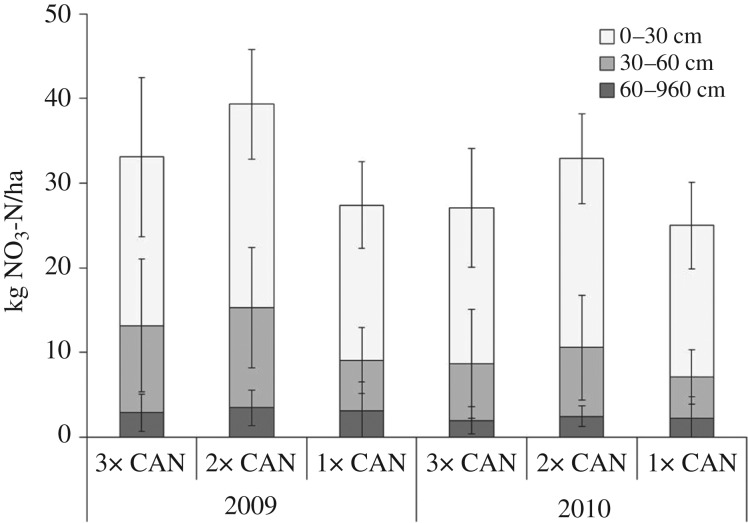
NO_3_^−^-N after harvest in the soil layers 0–30, 30–60 and 60–90 cm of the split (3×, 2×) and single (1×) applications of calcium ammonium nitrate (CAN) in 2009 and 2010. Bars show s.d.

The inorganic N after harvest in 2007 and 2008 accounted for <20 kg N/ha in the top soil (0–30 cm) and <10 kg N/ha in the 30–60 cm layer, and showed no significant differences between the fertilization treatments in either year (data not shown). In 2009 and 2010, splitting of CAN had no effect on the content of residual inorganic N in the soil profile (Fig. 5).

**Fig. 6.**
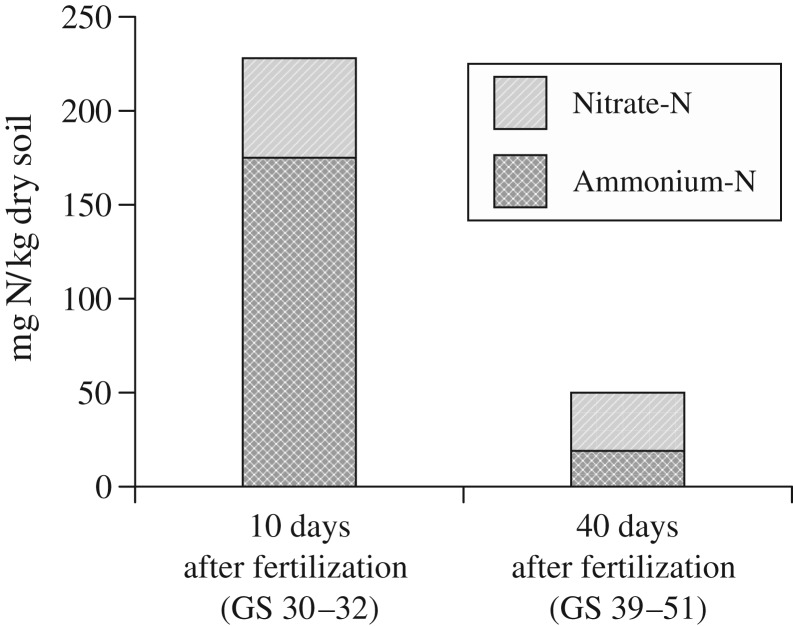
NH_4_^+^-N and NO_3_^−^-N in the placement band of urea ammonium nitrate solution (UAN) 10 and 40 days after fertilization in 2008.

The soil samples from the labelled placement bands 10 days after the application of UAN at GS 30–32 showed a concentration of 230 mg inorganic N/kg soil with a proportion of 0·77 NH_4_^+^-N in the year 2008 (Fig. 6). Forty days after the placed application, mineral N in the placement band was reduced to 50 mg N/kg soil with a proportion of 0·62 NO_3_^−^-N. The soil samples (0–30 cm) from the soil between the placements showed maximum soil values reaching 5 mg NH_4_^+^-N and 20 mg NO_3_^−^-N/kg soil during the vegetation period (data not shown). After harvest, the placed area was almost depleted and not significantly different to the other broadcast applied treatments.

## DISCUSSION

Grain yields between the treatments were not significantly different in any of the 4 years. It seems that a single N application between advanced tillering and beginning of stem elongation, either broadcast or placed, is sufficient to produce high yields on the current experimental sites. Furthermore, there were no significant differences between the fertilizer types CAN, urea and UAN. Consequently, there were no differences in fertilizer N use efficiency if comparing application techniques or fertilizer types. This finding can be attributed to the properties of the experimental sites. Luvisols with loamy texture are characterized by high fertility and high N mineralization potential, enabling winter wheat plants to compensate temporal periods of N deficiency caused by insufficient fertilizer N supply.

Among the other factors affecting yield, weather is the most important variable followed by the total amount of applied N, N application timings and technique (Fischbeck *et al.* 1990; Alcoz *et al.* 1993; Maidl *et al.* 1996). In 3 of the 4 years of the field experiments (2007, 2009, 2010) April was dry and relatively warm. Therefore, it was thought that N fertilization applied during this period would not be as effective as a split application with the first N fertilization already applied in March. Winter wheat plants suffering from N deficiency during stem elongation should develop a smaller number of grains/ear, thus resulting in grain yield reduction. Nevertheless, plants were able to overcome temporary N deficiency as well as drought stress without any negative effect on grain yield. This might have been the effect of high N mineralization potential and water holding capacity of the soils on the experimental sites; however, it showed the potential of modern varieties to compensate for intermediate shortcomings in nutrients and water.

The total amount of N supplied is more important for yield and quality of cereals than split applications (Müller *et al.* 1991). Obviously, the effect of different application times becomes more evident only with insufficient N supply. In the current experiments, there were no negative effects of single N applications on yield and quality, because total N supply was adapted to the N demand of the crops (Düngeverordnung 2009), even though N deficiency symptoms were sometimes visible in winter wheat plants before the N application at GS 27–32. Obviously, the winter wheat plants were able to overcome temporary N deficiency situations without negative effects on further growth and yield. Similar results were found by Boelcke (2001), Kücke (2001*a*) and Sommer (2000) with placed N applications.

The single broadcast application did not lead to lodging of winter wheat plants in any of the 4 years, even though no growth regulators were applied by farmers. Lodging should be particularly critical for distinct density types of wheat cultivars if the total amount of N is applied in a single dose. Nevertheless, although some winter wheat cultivars used in the field experiments tended to be density types, no lodging was visible. Obviously, modern winter wheat varieties characterized by high stem strength and reduced shoot length exhibit a decreased disposition to lodging.

No significant differences in grain crude protein were observed between treatments, except in 2010. The same results were found by Kücke (2001*b*). These findings are, however, in contrast to results of Boelcke (2001), who reported lower crude protein contents after a single placed application. Consequently, Boelcke (2001) recommended an application of N at GS 49–65 in order to obtain a higher crude protein content. In 2010, crude protein contents were exceptionally high in all treatments indicating a high N-uptake particularly during the latest stage, thus, determining protein storage. Under these conditions a late split application of N might promote protein storage as shown by the higher protein content in the 3×CAN treatment compared to the 1×CAN treatment. The high protein content of the single application of urea and UAN solution in 2010 can be explained by reduced mineralization of these fertilizers due to severe dryness in spring inducing a better N availability during protein storage.

Differences in the general crude protein level, due to year and site, might also explain the differences between independent investigations.

In contrast to the current results, slight differences between the efficiency of the N fertilizer type urea and CAN were found by Czauderna (1992) and Weimar (2001). One might assume that N fertilization with urea causes lower crude protein when surface applied during drought and high temperatures. Under these conditions gaseous N losses may reduce the effective N amount supplied.

The differences in mean crude protein in the individual years were partly explained by weather conditions during head emergence and flowering of winter wheat. For example, the relatively high crude protein content in 2007 can be explained by above average temperatures and rainfall during these late developmental stages. However, there seems to be a negative feedback concerning grain yield and crude protein content. As mentioned above, in 2008, the year with highest grain yield (9·4 t DM/ha), crude protein was lowest (11·9%). This might be due to a ‘dilution effect’ as a consequence of optimal growth conditions during tillering and stem elongation, promoting the formation of yield components. Under these conditions, endosperm low in protein is enlarged relative to the germ with high protein content. However, if the yield components ‘ears/m^2^’ and ‘number of grains/ear’ are reduced, probably as a result of spring drought, a high N availability during grain-filling, depending on N uptake and remobilization of N, might result in higher crude protein levels.

In wheat, the first N application is traditionally given at tillering in order to support the number of tillers/m^2^. Nitrogen has to be applied at the beginning of tillering around GS 21–25, particularly for plant stand density types, whereas an application at GS 31 should support the number of grains/ear and diminish the reduction of ears/m^2^ (Maidl *et al.* 1998). In the present study, in 2007 and in 2008 no significant differences in the number of ears/m^2^ between treatments or cultivars were detected. The reason may be a sufficient N supply in the soil during tillering. Sommer (2000) also found no differences between a single placed N application and a split application, whereas Kücke (2001*b*) reported higher numbers of ears/m^2^ after a single placed application. In general, plants are able to balance a reduced number of ears/m^2^. When ears/m^2^ are reduced the number of grains/ear and the thousand grain weight are relatively higher (Niehoff 1978). In 2007, a higher number of grains/ear for the single broadcast application of urea and the single placed application of UAN solution were measured, although April 2007 was extremely dry. It can be assumed that plant development was reduced by drought and the late N application became effective only after rainfall at the beginning of May.

The thousand grain weight depends on the availability of assimilates, which obviously was not limited in 2008. Kücke (2001*a*) also found no significant differences between the thousand grain weights of a single placed application and a split application. The amount of assimilates is limited and thousand grain weight reduced only if drought appears during grain filling, causing the plants to mature earlier.

It might be argued that the risk of NO_3_^−^-leaching during the vegetation period is increased by a single broadcast application of N (Gerwing *et al.* 1979). This might be true for sandy or shallow silty soils, but it is unlikely for deep loamy soils (Beaudoin *et al.* 2005), especially under the climatic conditions of South-West Germany, where evapotranspiration is quite high during the growth period. The inorganic N after harvest accounted for *c.* 20 kg N/ha in the topsoil and for 5–10 kg N/ha in the 30–60 cm layer without significant differences between the fertilization treatments, indicating that no NO_3_^−^-leaching occurred during the vegetation period.

The soil samples from the labelled placement bands 10 days after the application of UAN at GS 30–32 showed a concentration of 230 mg mineral N/kg soil with a proportion of 77% NH_4_^+^ in 2008. According to Sommer (2005), a more beneficial plant morphology compared to conventionally fertilized plants is an advantage of the placed application of NH_4_^+^ fertilizer. Zhang & Barber (1993) showed a linear increase in root length density and root surface within the diffusion zone of banded ammonium sulphate depending on the amount supplied. However, in the current study no visual differences between placed and conventionally fertilized plants were found. Additionally, no aggregation of roots around the placed area was visible when digging in the fertilizer bands. This might indicate that N nutrition of plants was not NH_4_^+^-dominated. This assumption goes along with Menge-Hartmann & Schittenhelm (2008) who found slight physiological reactions typical for NH_4_^+^-nutrition during stem elongation, but no differences between NH_4_^+^- and NO_3_^−^-fertilization at later stages of spring wheat development.

The lack of root aggregation around the placed area in the top soil can be advantageous to overcome drought compared to root aggregation in a dry topsoil. A reasonable method to tackle this problem and to maintain a high grain yield compared to broadcast application is to place N fertilizers in deeper soil layers, as was shown by Janzen *et al.* (1990). In soils with low N mineralization potential roots will exploit this nutrient patch, since N (mainly NO_3_^−^) triggers root formation towards high concentrations (Drew 1975; Forde & Lorenzo 2001) and thereby enlarges root to shoot ratio (Marschner 1995).

## CONCLUSION

The current results have shown that a reduction in the number of N applications to winter wheat with a late first application can be recommended for the climatic conditions of South-West Germany on medium to heavy textured deeply developed soils. If crude protein contents are negligible, i.e. in ethanol-wheat production, a single application is sufficient.

The findings strongly call for further investigations under different climatic conditions also including other crop species. The worldwide use of split applications of N fertilizer seems to be built at least partly on obsolete assumptions developed under conditions that are no longer valid, e.g. more flexible varieties and an overall higher level of fertilization compared to decades ago.

Most of the participating farmers have already adopted the reduction in the number of doses from three to two or even single application.

The authors thank the participating farmers for their intense cooperation and Mr. Scott Demyan for improving the English. We also thank the anonymous reviewers for their valuable suggestions. The experiments were supported by the Ministry of Rural Areas and Consumer Protection of the federal state of Baden-Württemberg, Germany.
